# Systematic review of products with potential application for use in the control of *Campylobacter* spp. in organic and free-range broilers

**DOI:** 10.1186/s13028-022-00644-z

**Published:** 2022-09-08

**Authors:** Brian Lassen, Birgitte Helwigh, Channie Kahl Petersen, Johanne Ellis-Iversen

**Affiliations:** 1 Research Group for Foodborne Pathogens and Epidemiology, National Food Institute, Technical University of Denmark, 2800 Kgs. Lyngby, Denmark; 2 Research Group for Global Capacity Building, National Food Institute, Technical University of Denmark, 2800 Kgs. Lyngby, Denmark; 3 Danish Veterinary and Food Administration, 2600 Glostrup, Denmark

**Keywords:** Disease prevention, Feed additives, Feed materials, Food safety, Interventions, Poultry, Public health, Zoonoses

## Abstract

*Campylobacter* spp. are some of the most important food-borne zoonoses in Europe and broiler meat is considered the main source of *Campylobacter* infections. Organic and free-range broilers have access to outdoor reservoirs of *Campylobacter* and are more frequently infected at slaughter than the conventional broiler flocks. Limitations to biosecurity and treatment options in these production types calls for additional solutions. This review examines intervention methods with sufficient strength and quality, which are able to reduce the load of *Campylobacter* safely and efficiently and discuss their applicability in organic and free-range broiler production. Four different products passed the inclusion criteria and their quality examined: ferric tyrosine chelate, a prebiotic fermentation product of *Saccharomyces cerevisiae*, short-chain fatty acid butyrate coated on microbeads added to feed, and a mix of organic acids added to the drinking water. Though potential candidates for reducing *Campylobacter* in broilers were identified, there is a lack of large scale intervention studies that demonstrate an effect under field conditions of a free-range broiler production.

**Supplementary Information:**

The online version contains supplementary material available at 10.1186/s13028-022-00644-z.

## Background

The zoonotic pathogen *Campylobacter* spp. is one of the most frequent causes of food-borne diarrhoeal diseases in the world [1], including Europe [2]. Broiler meat is considered the largest single source of campylobacteriosis in humans [3] and naïve flocks of broilers acquire *Campylobacter* infection from reservoirs in the farm environment [4].

In the conventional broiler production system, it is possible to reduce the risk of birds becoming infected with *Campylobacter* by controlling the environment of the birds by implementing biosecurity measures and separating the birds from the outdoors environment. In the production of organic and free-range broilers, the broilers have access to the outdoors, which exclude most options for strict biosecurity. In the European Union (EU), organic broilers are by definition free-range, and are bound by additional rules of production [5]. The frequency of flocks becoming infected with *Campylobacter* before slaughter is 2–3 times higher in organic flocks compared to conventionally raised broilers [6]. Though fewer organic broilers are produced than conventional broilers today, the demand for organic broilers is increasing. Between 2012 and 2019 the number of registered organic broilers doubled in the EU [7].

The EU aims to reduce the public health risk attributed to campylobacteriosis, by reducing the number of colony forming units (CFU) found on broilers entering the slaughterhouse [3]. For this reason, there is an interest in reducing infections with *Campylobacter* spp. in the flocks, before the broilers reach the slaughterhouse. Achieving this aim is especially challenging to producers of free-range broilers, due to (1) the broilers exposure to *Campylobacter* in the farm environment, (2) longer exposure times given organic chickens live longer than conventional chickens and (3) organic farms have more legal restrictions in their use of various additives, treatments and disinfectants [5, 8, 9].

Effect of treatments, disinfectants or additives on infection are ideally measured by intervention studies with the specific purpose to test the effectiveness of the preventive strategy. There have been a number of reviews which examine the status of the different interventions and their ability to reduce the presence of *Campylobacter* spp. in live chicken and in vitro [e.g. 10–12]. The reviews present excellent summaries of the existing research, but are not designed to address the applicability to specific production systems or the strength and reliability of the evidence. To our knowledge, it has not been attempted to review the quality and strength of evidence of the interventions of applicable products that have the potential to reduce the presence of *Campylobacter* spp., in the production of organic and free-range broilers.

In this review, we assess the value of studies from the last 10 years of peer-reviewed research to identify products with the potential to reduce the concentration (log10 CFU/g) of *Campylobacter* spp. in faeces and/or caecal contents in a free-range broiler production setting and evaluated the quality of the found evidence.

### Search strategy

Search strings were designed according to the research question, with the purpose of returning research articles that described the efficacy of interventions administrated to reduce the concentration of *Campylobacter* spp. in broilers. The search was carried out using DTU Findit search tool, which is operated and developed locally by DTU Library [13]. The data providers of DTU Findit included arXiv.org Eprint Archive, CrossRef, PubMed, Scopus, Web of Science, major publishers and universities [14]. The identification of articles was carried out from November to December 2018, and updated in February and August 2020.

The search terms included different types of intervention strategies, *Campylobacter* and broiler production. The full list of search terms is listed in Additional file 1. The articles included in the study were limited to research articles in English starting from 2010. Additional research articles were identified by reading literature reviews on intervention methods applied in the control of *Campylobacter* spp. in chicken [10–12, 15–20].

In the screening of titles and abstracts, duplicates and studies not matching our target criteria, were excluded [21]. The articles were entered in Excel (Microsoft Office Professional Plus 2016) and predefined descriptors for each article were added by screening the contents of the articles (Additional file 2). The descriptors were used in the remaining screening steps and evaluation of the quality of the study. The descriptors were used to identify and remove articles that did not meet criteria for intervention studies that would reduce *Campylobacter* concentrations sufficiently in broiler production with available methods using stepwise exclusion.

Inclusion criteria were defined to retain peer-reviewed publications that presented a significant reduction in a free-range broiler production setting. The inclusion criteria were:

- The study included and clearly described a control group.
- The effect was demonstrated on live chicken (in vivo).
- The age of the chicken at the end of the study matched that of slaughtered broilers.
- The effect of the tested product was significant (≤ 0.05) at the time of slaughter.
- The method tested was applicable in a free-range production system, i.e. the intervention logically can work under continuous exposure to different *Campylobacter* species naturally occurring in the outdoor environment.
- The intervention was a developed product.
- The reported reduction effect was large enough to have an impact to public health i.e. a reduction of ≥ 2 log10 CFU *Campylobacter.* This reduction was estimated to reduce the relative risk of campylobacteriosis attributable to the consumption of broiler meat with 42% (95% CI 11–75%) [22]).

The remaining articles were considered eligible and were evaluated for the quality of the evidence.

### Rating the quality of evidence

The confidence in the evidence from the included articles were evaluated based on GRADE guidelines [23]. The GRADE guidelines are a system designed for a transparent rating of the evaluation of the quality of evidence in reviews and offer guidelines for grading the strength of recommendations. In the evaluation each outcome of interest is determined by a specified effect. The outcome of this study is the reduction of *Campylobacter* spp. in broilers at the time of slaughter estimated by the effect on the concentration (log10 cfu/g) in the faeces and/or caecal contents.

Randomised trials started with a rating of 4 and observational studies were given a rating of 2, as randomization is a fundamental element for reducing bias when testing different treatments. The next rating was based on the magnitude of effect in the studies and for the demonstration of a dose dependent effect. These attributes were able to increase the rating by 1 or 2 points. Studies including risk of bias (absence of methods that control introducing bias), inconsistency of results (agreement across studies), indirectness of evidence (representativeness of test group and/or outcomes), imprecision of results (the confidence in the effect) and publication bias (number of studies and funding) were used to decrease the rating 1–2 points each (− 1 = serious, − 2 = very serious). The criteria used for adjusting the ratings are presented in Additional file 3. The final rating represents the level of confidence in the study on a scale from 4 (high), 3 (moderate), 2 (low), 1 (very low), or 0 (none).

A high level of confidence reflects a high confidence in the result presented in the study, and that the study result is close to the true level of the effect [24]. A moderate level of confidence level reflects a moderate confidence in the estimate, and that the true estimate is likely to be close to what is presented, but possibly could be substantially different. Low level of confidence reflects a limited confidence in the measured effect, and that the true effect possibly is substantially different. A very low level of confidence reflects a low confidence in the effect presented, and that the true effect is likely to be substantially different.

In addition to the GRADE guidelines, we also upgraded the quality, if the study was carried out under field conditions and was a free-range production. Field conditions were defined as: broilers raised in a broiler production facility under the normal production cycle and managed by the farmer.

## Review

**Fig. 1:**
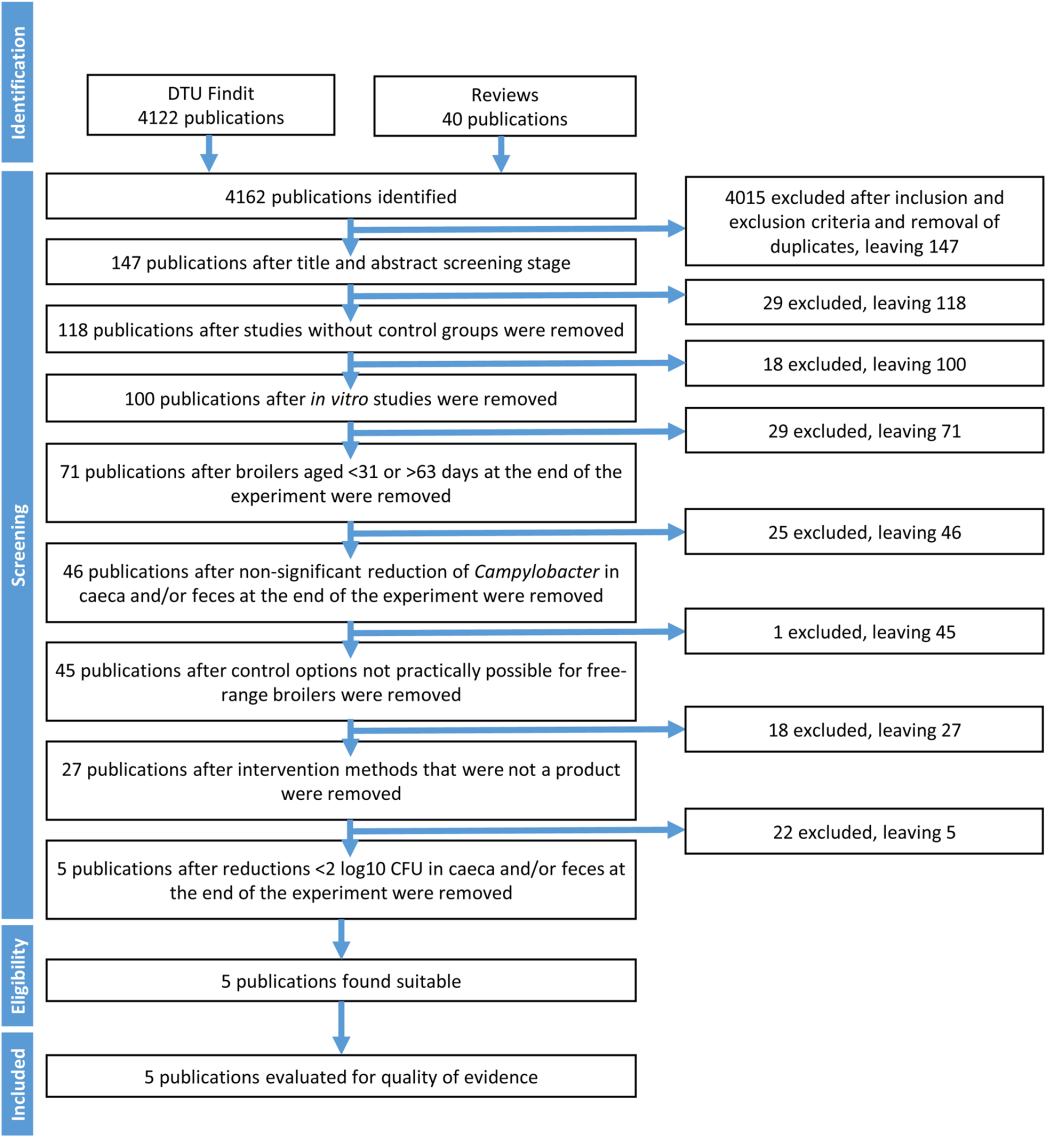
PRISMA flow chart of the review process

The steps in the literature review are shown in Fig. 1 and the stepwise removal is shown in Additional file 4. The effect of studies excluded in the final step of the screening process are listed in Additional file 5. Five articles [25–29] were retained and some of these studies had several interventions. We included Additional file 5 to show studies with < 2 log10 units reduction for readers interested in a list of interventions with a lower effect, but tested under conditions close to free-range broiler production. Of the six interventions that demonstrated more than a 2 log10 CFU reduction of *Campylobacter* spp. in the caeca or faeces of individual chickens, three were a non-classified ferric tyrosine chelate (FTC) feed additive, two were short-chain fatty acids in either feed or water, and one intervention was classified as a prebiotic feed additive (Table 1).

**Table 1:** Information extracted from five studies testing the effect of four products in reducing the concentration of *Campylobacter* in free-range broilers

Article	[27]	[28]	[29]	[26]	[26]	[25]
Composition (product name)	Ferric tyrosine chelate (TYPLEX® Chelate)	Ferric tyrosine chelate (TYPLEX® Chelate)	Ferric tyrosine chelate (TYPLEX® Chelate)	*Saccharomyces cerevisiae* fermentate (Original XPC®)	Butyrate coated on microbeads (Adimix® Precision)	Formic acid, acetic acid, propionic acid and sorbic acid (Selko® 4Health)
Type of product	Not classified	Not classified	Not classified	Prebiotic	Short-chain fatty acid	Short-chain fatty acids
Administration	Feed additive	Feed additive	Feed additive	Feed additive	Feed additive	Water additive
Randomization described	Yes	Yes	Yes	Yes	Yes	No
Blinding used	No	No	No	No	No	No
Establishment baseline infection status prior to intervention	No	No	Yes	Yes	Yes	No
Passive infection of broilers via the environment	Yes	Yes	Yes	No	No	Yes
Field study conditions	No	No	No	No	No	Yes
Country of study	United Kingdom	Scotland	Greece	France	France	Germany
Control group (N)	5	10	16	44	44	5
Intervention group (N)	5	10	16	15	15	4–5
Repeats (N)	6	10	6	1	1	3

None of the screened studies tested the effectiveness of a compound to reduce *Campylobacter* spp. in chicken under field conditions resembling free-range broiler production, and only one study described conditions that were similar to commercial indoor broiler production [25].

### Quality of evidence

The GRADE evaluation of the five studies retained after the screening process is presented in Table 2. Studies that tested FTC scored between very low and high. The confidence in the effect of FTC was scored as moderate. Increasing the confidence in the effect of FTC, was the use of randomization and the demonstration of a dose dependent effect in the studies. The studies on FTC all tested the same three doses of the product, and two of the studies were able to demonstrate a dose dependent effect. Only the effects of the two largest doses were ≥ 2 log10 cfu/g and were included in the screening process (Fig. 1). Subtracting from the quality of the study was that none of the studies testing FTC matched our definition of a field study (indirectness). In addition, publication bias could not be excluded, as the producer of the product had funded all three studies. One study did not provide exact estimates of the concentrations of *Campylobacter* from the caecal samples and was given a lower score for imprecision.

**Table 2:** GRADE evidence profile: reduction of concentration of *Campylobacter* spp. in broilers at slaughter

Quality assessment						Summary of findings			
Studies (design)	Limitations	Inconsistency	Indirectness	Imprecision	Publication bias	Control (log10 cfu/g ± SD)	Treated (log10 cfu/g ± SD)	Reduction (log10 cfu/g)	Quality
Ferric tyrosine chelate, 0.05 g/kg, slaughter at day 42 (2 RCT)									
[27]	No serious limitations	No serious inconsistency	Serious indirectness (not field study)	No serious imprecision	Serious publication bias (funding)	4.799 ± NA	2.399^a^ ± NA	2.400	⊕⊕⊕⊕ High
[28]	No serious limitations	No serious inconsistency	Serious indirectness (not field study)	No serious imprecision	Serious publication bias (funding)	5.86 ± NA	3.81 ± NA	2.05	⊕⊕⊕O Moderate
Ferric tyrosine chelate, 0.20 g/kg, slaughter at day 42 (3 RCT)									
[27]	No serious limitations	No serious inconsistency	Serious indirectness (not field study)	No serious imprecision	Serious publication bias (funding)	4.799 ± NA	1.681 ± NA	3.118	⊕⊕⊕⊕ High
[28]	No serious limitations	No serious inconsistency	Serious indirectness (not field study)	No serious imprecision	Serious publication bias (funding)	5.86 ± NA	3.74 ± NA	2.12	⊕⊕⊕O Moderate
[29]	No serious limitations	No serious inconsistency	Serious indirectness (not field study)	Serious imprecision (results imprecise)	Serious publication bias (funding)	NA	NA	2	⊕OOO Very low
*Saccharomyces cerevisiae* fermentate, 0.125% (wt/wt), slaughter day 42 (1 RCT)									
[26]	No serious limitations	No serious inconsistency	Very serious indirectness (not field study + oral inoculation)	No serious imprecision	No publication bias found	6.29 ± 2.20	3.13 ± 2.87	3.17	⊕⊕⊕O Moderate
Butyrate coated on microbeads, 0.300% (wt/wt), slaughter day 42 (1 RCT)									
[26]	No serious limitations	Serious inconsistency (because of lack of consensus)	Very serious indirectness (not field study + oral inoculation)	No serious imprecision	No publication bias found	6.29 ± 2.20	4.16 ± 2.67	2.13	⊕⊕OO Low
Formic acid, acetic acid, propionic acid and sorbic acid, 0.075%, slaughter at day 42 (1 OBS)									
[25]	No serious limitations	No serious inconsistency	No serious indirectness	No serious imprecision	Serious publication bias (funding)	6.25 ± 0.93	1.99 ± 1.62	4.26	⊕⊕OO Low

*NA* not available

Guyard-Nicodème et al. [26] tested 12 commercial products of which two met the criteria in this review. The prebiotic fermentate of the yeast *Saccharomyces cerevisiae* was evaluated to have a moderate quality of evidence, and butyrate coated on microbeads was evaluated to have a low quality of evidence. Increasing the quality of the study was the use of randomization and the demonstration of large effects. The large effect was demonstrated by a reduction in the concentration of *Campylobacter* in the broilers when slaughtered 42 days old by more than 3 log10 cfu/g when *Saccharomyces cerevisiae* was added to the feed. The large effect of butyrate coated on microbeads added to the feed was demonstrated by significantly reducing the concentration of *Campylobacter* in the broilers for the entirety of the experimental period (samples taken 14, 35, and 42 days of age). Subtracting from the quality of the studies was that the experimental design did not describe field conditions and individual chickens were inoculated orally with *Campylobacter* rather than passive transfer (indirectness). Butyrate coated on microbeads got a lower score for inconsistency due to the lack of consensus supporting an effect.

The addition of several short-chain fatty acids in the drinking water was evaluated to have a low quality of evidence. Increasing the quality of the study was a large effect. Subtracting from the quality of the study was the lack of randomization described in its design, giving it a lower initial score. Further subtracting from the quality of study was that publication bias could not be excluded due to funding from the producer of the tested product. The study was carried out under field conditions, which was accounted for by not subtracting from the score due to indirectness as in the other studies.

### Discussion

Reduction of *Campylobacter* in broilers using different products added to feed or drinking water has been thoroughly researched over the last decade. There are products that show an effect, but an overview of the quality of the studies, confidence in the outputs and synergy in results for the same products was lacking. This study was designed to address this gap. Our screening criteria were designed to find products with sufficient evidence of reducing the concentration of *Campylobacter* in free-range broilers sufficiently enough to obtain a positive impact on public health. Only a few products had progressed beyond laboratory trials, to experimental trials and into a product available to the farmer. Furthermore, very few studies demonstrated the sufficient effect ≥ 2 log10 units reduction in the concentration of *Campylobacter* in broilers at the time of slaughter.

It was only possible to find one study that met the study’s definition of a field trial, and this study used a conventional broiler production [25]. No intervention studies tested under the production of organic and free-range broilers remained after our screening steps. However, two of the excluded studies had tested feed additives on broilers with outdoor access, but these studies were excluded because the observed *Campylobacter* reductions were not significant in faeces or caecum at slaughter [30, 31]. The review demonstrated that there is a need for field trials, designed rigorously enough to report a significant reduction in the concentration of *Campylobacter* in the faeces of free-range broilers at the time of slaughter.

The screening criteria excluded a number of interventions that have otherwise showed promising results experimentally, such as bacteriophages and vaccines [12, 19, 22]. These interventions may or may not advance to demonstrate sufficient and reproducible effects in field trials on both conventional and organic/free-range broiler farms in years to come. Currently these options are not available to the farmer and demonstration of reproducible results under field conditions are needed [32].

This review aimed to identify the products with the strongest evidence of working in a production setting resembling free-range broiler farms. We added further detail by assessing the confidence in the evidence based on the GRADE guidelines. The GRADE guidelines are used to evaluate a body of evidence, or individual studies, to be able to give reasoned recommendations [23]. Though the GRADE guidelines is a systematic approach, it is still an evaluation and thus includes subjective elements. In this evaluation, we placed less weight on study limitations, because certain compromises are expected for intervention studies under field/near-field conditions, but were more critical on the potential for publication bias.

FTC was the product with the highest overall quality of evidence. The feed additive is a constellation of three tyrosine amino acids around an Iron (III)-ion [33]. The exact mechanism of FTC is unclear, but it is thought to block outer membrane proteins of *Campylobacter*, inhibiting the bacteria from binding to host cells and creating a biofilm [27].

All three studies testing the effect of FTC were conducted using passive transfer of *Campylobacter* from the environment to the birds; i.e. from housing a flock where there had previously been infected birds [27], spreading manure from infected birds [29], to creating bacterial suspensions mixed with droppings strategically placed near a feeder [28]. For results to be applicable in practice the use of horizontal infections in intervention studies is important, as using inoculations can result in a different result in concentrations of *Campylobacter* at the age of slaughter in the longer-living free-range broilers [34].

The overall confidence in FTC was evaluated to be moderate, assessing that the results reflected a true estimate, but that it is possible, that it is substantially different. This was based on a relatively conservative evaluation of the confidence in the study, by not excluding the possibility of publication bias and subtracting for indirectness for not being a field study. The study by Skoufos et al. [29] was evaluated lower than the other studies as exact data on the reduction was lacking, and the study was unable to replicate the dose dependent effect seen in the other studies. This may be explained by a higher initial concentration of *Campylobacter* in the group pens of broilers that were administered the lowest dose (0.05 g/kg) FTC, and a lower initial concentration in the group administered 0.20 g/kg FTC compared to the control group.

Additional investigations support the effect of FTC. EFSA evaluated the effect of FTC in two reports. One scientific report examined 5 in vitro and 6 in vivo studies and concluded that at least 1 log10 CFU reduction of *Campylobacter* in broilers is achievable with 0.02 g/kg FTC [33]. In the updated review of the current different control options for *Campylobacter* in broilers at primary production level, FTC was evaluated to be one of three feed additives to have a sufficiently large effect on the reduction of the relative risk of human campylobacteriosis in the EU [22].

The reproducibility or consistency between studies and researchers in showing an effect of FTC is increasing the confidence that application of this product may result in a reduction in *Campylobacter* in poultry. However, no outdoor field trials of the product were found, and it is unknown whether the environment, e.g. exposure to changing temperatures, different microorganisms, UV-light, rain etc., influence the effect of the compound.

The study by Guyard-Nicodème et al. [26] investigated the efficacy of 12 feed additives on reducing *Campylobacter* in live broilers when the birds were 14, 35 and 42 days of age, using the same experimental setup. The latter two periods of slaughter mimic thinning, which is similar to the practice in some free-range broiler productions due to the slower growth and uneven size of the birds. *Saccharomyces cerevisiae* and butyrate coated on microbeads were included.

*Saccharomyces cerevisiae* is a yeast used in baking and wine production. It is used as a probiotic, stimulating the gut microbiome of the bird, and promoting bacteria that may be antagonistic to *Campylobacter* [35]. *Saccharomyces cerevisiae* together with other probiotics has been demonstrated to reduce *Campylobacter* in one field study [36]. The *Saccharomyces cerevisiae* strain *boulardii* has been used to successfully reduce the concentration of *Campylobacter* in several studies [37–39]. There are restrictions to what can be added to animals feed in organic production. According to the EU law on organic production *Saccharomyces cerevisiae* is approved as a substance that can be used in animal nutrition in certain circumstances [9]. The product tested by Guyard-Nicodème et al. [26] is a fermentate produced by *Saccharomyces cerevisiae* and according to the producer contain no live organisms. The tested product is not designed for use in organic production, but the same feed additive is also produced in a formulation made for use in organic production.

Butyrate is a short-chain fatty acid and is the salt of butyric acid. Butyric acid is produced by bacteria in the gut of broilers [40]. Short-chain fatty acids are thought to have a bactericidal effect by lowering the pH in the digestive system of the chicken [41]. In addition, some short-chain fatty acids may diffuse across a bacterial membrane and dissociate protons in the cytoplasm, harming the bacteria [42]. The product using butyrate coated on microbeads was the only of 12 products that lowered the concentration of *Campylobacter* in broilers throughout the study, demonstrating a consistent effect. A second product with butyrate coating on microbeads was tested in the same study, but at 1/3 (0.1% wt/wt) of the concentration that demonstrated an effect in the study by Guyard-Nicodème et al. [26]. The lower concentration was able to reduce the concentration of *Campylobacter* only when the broilers were 14 days of age. Another study using 0.1% wt/wt butyrate was not able to demonstrate a reduction of *Campylobacter* in broilers [34], but even lower concentrations of 0.05% wt/wt have demonstrated an effect [43]. Combining butyric acid (salts and esters of butyric acid) glycerides with protected organic acids in the feed, has demonstrated a reduction of *Campylobacter* in the caecum at 42 days of age [44].

Because butyrate is produced naturally by obligate anaerobic bacteria in the mammalian gut, this organic acid may be possible to use in organic broiler production [45]. However, the unknown properties of the microbeads encapsuling the acid for the identified product may limit its applicability for this production type.

The only field study included in this review used a product that added a mix of organic acids in the broilers drinking water: formic acid, acetic acid, propionic acid and sorbic acid. Drinking water and surface water is a known risk factor for the spread of *Campylobacter* in broiler flocks [46]. The application of organic acids to the drinking water may thus potentially reduce the presence of *Campylobacter* in both the environment and in the bird. No other studies tested the same product, but other combinations of organic acids in drinking water have demonstrated a reduction of *Campylobacter* in broilers [41, 47–50]. The EU law on organic production list all the acids in the tested product by Jansen et al. [25] on the list of allowed zoo-technical feed additives [9]. The main reason the confidence in the study was not evaluated higher was because no randomization procedure could be identified.

The review succeeded in finding potential candidates for controlling *Campylobacter* in broilers, some of which may be accepted for use in organic production. There is a need for studies that test the effect of interventions on free-range broilers exposed to *Campylobacter* via horizontal transfer with clear descriptions of randomization, blinding, description of baseline infection status where applicable and ensuring all results including predictions of uncertainties are made available. Though the findings presented were targeted at finding *Campylobacter*-control-options for free-range broilers, the studies found are also applicable to conventional broiler production.

## Conclusion

Four products were identified with potential of reducing the concentration of *Campylobacter* at the time of slaughter in broilers. The products with the highest confidence in the evidence were in descending order: ferric tyrosine chelate in feed, *Saccharomyces cerevisiae* in feed, butyrate coated on microbeads in feed, and organic acids in drinking water (formic acid, acetic acid, propionic acid and sorbic acid). None of the intervention studies were performed under field conditions resembling organic and free-range broiler production, but we identified potential eligible candidates that could be tested in future trials.

## Supplementary Information

**Additional file 1.** Search strings used in DTU Findit search too to find publications.

**Additional file 2.** Descriptors used to evaluate the articles.

**Additional file 3.** Defined criteria based on GRADE guidelines used for quality assessment of studies.

**Additional file 4.** Full data of selection process. Step by step exclusion of the identified article. The steps corresponds to the steps in Fig. 1.

**Additional file 5.** Reduction of *Campylobacter*. List of articles before the final step in the review process (Fig. 1), the tested interventions and the effect of the interventions.

## Data Availability

All data generated or analysed during this study are included in this published article and its Additional files.

## Abbreviations

CFU: Colony forming units
DTU: Danmarks Tekniske Universitet [Technical University of Denmark]
ECDC: European Centre for Disease Prevention and Control
EFTC: Ferric tyrosine chelate
EFSA: European Food Safety Authority
EU: European Union
GRADE: Grading of Recommendations Assessment, Development, and Evaluation
UV: Ultra-violet
