# Supplementary material for: Systematic review of products with potential application for use in the control of Campylobacter spp. in organic and free-range broilers
Source: Acta Vet Scand. 2022 Sep 8;64:24. doi: 10.1186/s13028-022-00644-z (PMC9461118; doi:10.1186/s13028-022-00644-z)
Supplement: Supplementary file 1 — Additional file 1. Search strings used in DTU Findit search too to find publications. [file 13028_2022_644_MOESM1_ESM.docx]

**Additional file 1. Search strings used in DTU Findit search too to find publications**

| Search string |
| --- |
| Campylobacter control broiler production AND ("feed additives" OR probiotics) |
| Campylobacter control broiler production AND feeding practices |
| Campylobacter control broiler production farming practices |
| Campylobacter control broiler production biosecurity |
| Campylobacter broiler biosecurity |
| Campylobacter broiler feed types/ Campylobacter control broiler feed types |
| Campylobacter broiler feed additives |
| Campylobacter broiler probiotics |
| Campylobacter broiler bacteria-phages |
| Campylobacter poultry bacteriophages |
| Campylobacter poultry farming practices |
| Campylobacter poultry feed additives |
| Campylobacter broiler feeding practices/Campylobacter poultry feeding practices |
| Campylobacter AND (chickens OR chicken OR broiler OR broilers OR poultry) AND biosecurity |
| Campylobacter AND (chickens OR chicken OR broiler OR broilers OR poultry) AND feed additives |
| Campylobacter AND (chickens OR chicken OR broiler OR broilers OR poultry) AND probiotics |
| Campylobacter AND (chickens OR chicken OR broiler OR broilers OR poultry) AND feed types |
| Campylobacter AND (chickens OR chicken OR broiler OR broilers OR poultry) AND farming practices |
| Campylobacter AND (chickens OR chicken OR broiler OR broilers OR poultry) AND feeding practices |
| Campylobacter AND (chickens OR chicken OR broiler OR broilers OR poultry) AND feed supplements |
| Campylobacter AND (chickens OR chicken OR broiler OR broilers OR poultry) AND phages |
| Campylobacter AND (chickens OR chicken OR broiler OR broilers OR poultry) AND risk factors |
| Campylobacter AND (chickens OR chicken OR broiler OR broilers OR poultry) AND interventions |
