# Supplementary material for: Systematic review of products with potential application for use in the control of Campylobacter spp. in organic and free-range broilers
Source: Acta Vet Scand. 2022 Sep 8;64:24. doi: 10.1186/s13028-022-00644-z (PMC9461118; doi:10.1186/s13028-022-00644-z)
Supplement: Supplementary file 2 — Additional file 2. Descriptors used to evaluate the articles. [file 13028_2022_644_MOESM2_ESM.docx]

**Additional file 2. Descriptors used to evaluate the articles**

| **Descriptor** | **Options** |
| --- | --- |
| Reference | Reference name |
| Author | First author last name |
| Year | Year of publication |
| Method 1 | Intervention group (feed additive, water additive, vaccine, other) |
| Method 2 | Type of intervention (bacteriophage, bacteriocin, probiotic, prebiotic, other) |
| Method 3 | Specific details (example: *Bifidobacterium longum*) |
| Line of evidence | Peer review, grey literature, company website |
| Type of study | Field, experiment, lab or model |
| Production type | Organic, free range, conventional, caged |
| Control group | Included a control group, a control group but no description of it, no control group |
| Aim of study | A description of the aim of the study, and where the study took place. |
| Campylobacter species | Describe species (*C. jejuni, C. coli,* other) |
| Campylobacter species | *Campylobacter* concentration tested before the intervention starts (yes/no) |
| Initial Campylobacter concentration | What is the concentration of *Campylobacter* at the start of the study (log10 CFU, not given) |
| Intervention method | Describe intervention methods (ex. Subcutaneously administrated vaccine administrated to 1 day old chicks). |
| Availability | (commercially available, created in a laboratory) |
| Application of the intervention | Where was the intervention applied? (drinking water, food, inoculation, vaccination of day old chicks etc.?) |
| Age at final effect testing | Age of the animals at end of the trial (days) |
| Sample description | Faeces (yes, no) |
| Statistical analysis | Statistical analysis (yes, no) |
| Geography | Where was the study carried out (Region or country) |
| Funding | (public, industry, product producer) |
| Who performed the study | (public, industry, product producer) |
| Who analysed the data/wrote the paper | (public, industry, product producer) |
| Significance of effect | (P-value) |
| Effect of each intervention | (Log10 CFU or % reduction in *Campylobacter*-positive chicken) |
