# Supplementary material for: Systematic review of products with potential application for use in the control of Campylobacter spp. in organic and free-range broilers
Source: Acta Vet Scand. 2022 Sep 8;64:24. doi: 10.1186/s13028-022-00644-z (PMC9461118; doi:10.1186/s13028-022-00644-z)
Supplement: Supplementary file 3 — Additional file 3. Defined criteria based on GRADE guidelines used for quality assessment of studies. [file 13028_2022_644_MOESM3_ESM.docx]

**Additional file 3. Defined criteria based on GRADE guidelines used for quality assessment of studies**

| **Adjustment** | **Criteria** | **Adjustment of rating** |
| --- | --- | --- |
| Higher if | Large effect   - ≥3 log10 CFU reduction - An effect was demonstrated over several sampling times spanning >14 days | +1 (one from list of criteria)  +2 (two from list of criteria) |
|  | Dose response | +1 |
|  |  |  |
| Lower if | Limitations   - Control groups were not described - Data was left out that could affect the evaluation of the effect - No baseline was established for the infection, when the intervention was applied after the birth of the animal - Not using blinding when administrating interventions individually | -1 (one from list of criteria)  -2 (two from list of criteria) |
|  | Inconsistency   - Other studies do not demonstrate an effect - More studies not demonstrating an effect than demonstrating an effect | -1 (one from list of criteria)  -2 (two from list of criteria) |
|  | Indirectness   - Chicken inoculated - Samples were not collected from individual chicken - Study not carried out under field conditions | -1 (one from list of criteria)  -2 (two from list of criteria) |
|  | Impression   - Specific results were not given - Uncertainties were not given | -1 (one from list of criteria)  -2 (two from list of criteria) |
|  | Publication bias   - Some of the authors were from the industry - The study was financed by the industry or interest groups - Negative studies were not published | -1 (one from list of criteria)  -2 (two from list of criteria) |
