# Supplementary material for: Systematic review of products with potential application for use in the control of Campylobacter spp. in organic and free-range broilers
Source: Acta Vet Scand. 2022 Sep 8;64:24. doi: 10.1186/s13028-022-00644-z (PMC9461118; doi:10.1186/s13028-022-00644-z)
Supplement: Supplementary file 4 — Additional file 4. Full data of selection process. Step by step exclusion of the identified article. The steps corresponds to the steps in Fig. 1. [file 13028_2022_644_MOESM4_ESM.docx]

**Additional file 4. Full data of selection process**

| **Step 1 (n=147)** | **Step 2 (n=118)** | **Step 3 (n=100)** |
| --- | --- | --- |
| Aguiar et al. 2013 | Aguiar et al. 2013 | Aguiar et al. 2013 |
| Annamalai et al. 2013 | Annamalai et al. 2013 | Annamalai et al. 2013 |
| Ansari-Lari, 2012 |  |  |
| Arsi et al. 2014 | Arsi et al. 2014 | Arsi et al. 2014 |
| Arsi, 2015 | Arsi, 2015 | Arsi, 2015 |
| Baffoni et al. 2017 | Baffoni et al. 2017 | Baffoni et al. 2017 |
| Bahrndorff et al. 2013 | Bahrndorff et al. 2013 | Bahrndorff et al. 2013 |
| Bahrndorff, 2015 | Balykina, 2019 | Balykina, 2019 |
| Balykina, 2019 |  |  |
| Battersby, 2017 |  |  |
| Bello et al. 2018 | Bello et al. 2018 |  |
| Branciari, 2016 | Branciari, 2016 | Branciari, 2016 |
| Buckley et al. 2010 | Buckley et al. 2010 | Buckley et al. 2010 |
| Bügener 2014 | Bügener 2014 | Bügener 2014 |
| Carvalho et al. 2010 | Carvalho et al. 2010 | Carvalho et al. 2010 |
| Cawthraw and Newell 2010 | Cawthraw and Newell 2010 | Cawthraw and Newell 2010 |
| Cean et al. 2015 | Cean et al. 2015 | Cean et al. 2015 |
| Chinivasagam et al, 2020 | Chinivasagam et al, 2020 | Chinivasagam et al, 2020 |
| Chintoan-Uta et al. 2016 | Chintoan-Uta et al. 2016 | Chintoan-Uta et al. 2016 |
| Chowdhury, 2012 |  |  |
| Chowdhury, 2013 |  |  |
| Clark et al. 2012 | Clark et al. 2012 | Clark et al. 2012 |
| Corrigan 2017 | Corrigan 2017 | Corrigan 2017 |
| Currie et al. 2018 | Currie et al. 2018 | Currie et al. 2018 |
| Dec et al. 2018 | Dec et al. 2018 |  |
| Diaz-Sanchez et al. 2015 |  |  |
| Ebrahimi et al. 2015 | Ebrahimi et al. 2015 | Ebrahimi et al. 2015 |
| EFSA Panel on Biological Hazards et al. 2020 | EFSA Panel on Biological Hazards et al. 2020 | EFSA Panel on Biological Hazards et al. 2020 |
| EFSA, 2019 |  |  |
| El-Ghany et al. 2015 | El-Ghany et al. 2015 | El-Ghany et al. 2015 |
| Ellis-Iversen, 2012 |  |  |
| Epps et al. 2015 | Epps et al. 2015 | Epps et al. 2015 |
| Firlieyanti et al. 2016 | Firlieyanti et al. 2016 |  |
| Froebel et al. 2019 | Froebel et al. 2019 | Froebel et al. 2019 |
| Ganan et al. 2013 | Ganan et al. 2013 |  |
| Garba, 2019 | Garba, 2019 | Garba, 2019 |
| Gaucher et al. 2015 | Gaucher et al. 2015 | Gaucher et al. 2015 |
| Ghareeb et al. 2012 | Ghareeb et al. 2012 | Ghareeb et al. 2012 |
| Gracia et al. 2016 A | Gracia et al. 2016 A | Gracia et al. 2016 A |
| Gracia et al. 2016 B | Gracia et al. 2016 B | Gracia et al. 2016 B |
| Grilli 2013 | Grilli 2013 | Grilli 2013 |
| Guyard-Nicodème et al. 2017 | Guyard-Nicodème et al. 2017 | Guyard-Nicodème et al. 2017 |
| Guyard-Nicodème et al. 2016 | Guyard-Nicodème et al. 2016 | Guyard-Nicodème et al. 2016 |
| Hammerl et al. 2014 | Hammerl et al. 2014 | Hammerl et al. 2014 |
| Hansson, 2010 |  |  |
| Haughton et al. 2013 | Haughton et al. 2013 | Haughton et al. 2013 |
| Henry, 2011 |  |  |
| Hermans et al. 2011 | Hermans et al. 2011 | Hermans et al. 2011 |
| Hermans et al. 2012 | Hermans et al. 2012 | Hermans et al. 2012 |
| Hermans et al. 2014 | Hermans et al. 2014 | Hermans et al. 2014 |
| Hodgins et al. 2015 | Hodgins et al. 2015 | Hodgins et al. 2015 |
| Hovorková & Skřivanová, 2015 | Hovorková & Skřivanová, 2015 | Hovorková & Skřivanová, 2015 |
| Huneay-Salaün et al. 2018 | Huneay-Salaün et al. 2018 | Huneay-Salaün et al. 2018 |
| Høg, 2016 |  |  |
| Jansen et al. 2014 | Jansen et al. 2014 | Jansen et al. 2014 |
| Johnson et al. 2015 | Johnson et al. 2015 | Johnson et al. 2015 |
| Jonsson, 2012 |  |  |
| Kahn et al. 2020 I | Kahn et al. 2020 I |  |
| Kahn et al. 2020 II |  |  |
| Karaffová et al. 2020 |  |  |
| Kelly et al. 2017 | Kelly et al. 2017 | Kelly et al. 2017 |
| Khan, 2019 | Khan, 2019 | Khan, 2019 |
| Khattak et al. 2018 | Khattak et al. 2018 | Khattak et al. 2018 |
| Kittler et al. 2014 | Kittler et al. 2014 | Kittler et al.. 2013 |
| Kittler et al.. 2013 | Kittler et al.. 2013 |  |
| Kobierecka et al. 2016 | Kobierecka et al. 2016 | Kobierecka et al. 2016 |
| Kobierecka et al. 2017 | Kobierecka et al. 2017 | Kobierecka et al. 2017 |
| Kurekci et al. 2014 | Kurekci et al. 2014 | Kurekci et al. 2014 |
| Łaniewski et al. 2014 | Łaniewski et al. 2014 | Łaniewski et al. 2014 |
| Laukava et al. 2017 | Laukava et al. 2017 | Laukava et al. 2017 |
| Layton et al. 2011 | Layton et al. 2011 | Layton et al. 2011 |
| Liu, 2018 | Liu, 2018 | Liu, 2018 |
| Lourenco, 2019 | Lourenco, 2019 | Lourenco, 2019 |
| Makavchik, 2019 |  |  |
| Mañes-Lázaro et al. 2017 | Mañes-Lázaro et al. 2017 | Mañes-Lázaro et al. 2017 |
| Mannelli, 2019 | Mannelli, 2019 |  |
| Massacci et al. 2019 | Massacci et al. 2019 | Massacci et al. 2019 |
| Massaoudi et al. 2012 | Massaoudi et al. 2012 |  |
| Metcalf et al. 2011 | Metcalf et al. 2011 | Metcalf et al. 2011 |
| Meunier et al. 2017 | Meunier et al. 2017 | Meunier et al. 2017 |
| Moen et al. 2012 | Moen et al. 2012 | Moen et al. 2012 |
| Molatová et al. 2011 | Molatová et al. 2011 | Molatová et al. 2011 |
| Molnár, 2014 | Molnár, 2014 | Molnár, 2014 |
| Mortada et al. 2020 | Mortada et al. 2020 | Mortada et al. 2020 |
| Nastasijevic et al. 2020 |  |  |
| Navarro et al. 2015 | Navarro et al. 2015 |  |
| Neal-McKinney et al. 2012 | Neal-McKinney et al. 2012 |  |
| Nishii et al. 2015 | Nishii et al. 2015 | Nishii et al. 2015 |
| Nishiyama et al. 2014 | Nishiyama et al. 2014 | Nishiyama et al. 2014 |
| Nothaft et al. 2016 | Nothaft et al. 2016 | Nothaft et al. 2016 |
| Nothaft et al. 2017 | Nothaft et al. 2017 | Nothaft et al. 2017 |
| Nowaczek, 2019 |  |  |
| Nuengjamnong & Luangtongkum 2014 | Nuengjamnong & Luangtongkum 2014 | Nuengjamnong & Luangtongkum 2014 |
| Ocejo et al. 2017 | Ocejo et al. 2017 | Ocejo et al. 2017 |
| Orquera et al. 2012 | Orquera et al. 2012 |  |
| Park et al. 2017 | Park et al. 2017 | Park et al. 2017 |
| Pitter 2018 |  |  |
| Pogacar, 2020 | Pogacar, 2020 |  |
| Prasai et al. 2016 | Prasai et al. 2016 | Prasai et al. 2016 |
| Ranjitkar, 2016 | Ranjitkar, 2016 | Ranjitkar, 2016 |
| Rashid et al. 2020 | Rashid et al. 2020 | Rashid et al. 2020 |
| Rezaei et al. 2015 | Rezaei et al. 2015 |  |
| Riazi et al. 2013 | Riazi et al. 2013 | Riazi et al. 2013 |
| Richards, 2019 | Richards, 2019 | Richards, 2019 |
| Robyn et al. 2013 | Robyn et al. 2013 | Robyn et al. 2013 |
| Saint-Cyr et al. 2017 | Saint-Cyr et al. 2017 | Saint-Cyr et al. 2017 |
| Salaheen, 2014 | Salaheen, 2014 |  |
| Sandberg, 2015 |  |  |
| Santini et al. 2010 | Santini et al. 2010 | Santini et al. 2010 |
| Sasaki, 2011 |  |  |
| Shrestha et al. 2017 | Shrestha et al. 2017 | Shrestha et al. 2017 |
| Sima et al. 2018 | Sima et al. 2018 | Sima et al. 2018 |
| Šimunović et al. 2020 | Šimunović et al. 2020 | Šimunović et al. 2020 |
| Schneitz and Hakkinen 2016 | Schneitz and Hakkinen 2016 | Schneitz and Hakkinen 2016 |
| Skoufos et al. 2019 | Skoufos et al. 2019 | Skoufos et al. 2019 |
| Skånseng et al. 2013 | Skånseng et al. 2013 | Skånseng et al. 2013 |
| Smialek et al. 2018 | Smialek et al. 2018 | Smialek et al. 2018 |
| Smith, 2016 |  |  |
| Sommer et al. 2016 A |  |  |
| Sommer et al. 2016 B | Sommer et al. 2016 B |  |
| Sommer, 2013 |  |  |
| Soro et al. 2020 |  |  |
| Svetoch et al. 2011 | Svetoch et al. 2011 | Svetoch et al. 2011 |
| Sweeney et al. 2017 | Sweeney et al. 2017 | Sweeney et al. 2017 |
| Szott et al. 2020 | Szott et al. 2020 | Szott et al. 2020 |
| Taha-Abdelaziz et al. 2019 | Taha-Abdelaziz et al. 2019 |  |
| Theoret et al. 2012 | Theoret et al. 2012 | Theoret et al. 2012 |
| Thépault et al. 2020 | Thépault et al. 2020 | Thépault et al. 2020 |
| Thibodeau et al. 2014 | Thibodeau et al. 2014 | Thibodeau et al. 2014 |
| Thibodeau et al. 2015 | Thibodeau et al. 2015 | Thibodeau et al. 2015 |
| Thomrongsuwannakij et al. 2016 | Thomrongsuwannakij et al. 2016 | Thomrongsuwannakij et al. 2016 |
| Thung et al. 2020 | Thung et al. 2020 |  |
| Torralbo, 2014 |  |  |
| Tsiouris, 2018 | Tsiouris, 2018 | Tsiouris, 2018 |
| Ushanov et al. 2020 |  |  |
| Valečková et al. 2020 | Valečková et al. 2020 | Valečková et al. 2020 |
| van Bunnik et al. 2012 | van Bunnik et al. 2012 | van Bunnik et al. 2012 |
| Vandeputte, 2019 | Vandeputte, 2019 | Vandeputte, 2019 |
| Visscher, 2019 | Visscher, 2019 | Visscher, 2019 |
| Wagenberg, 2016 |  |  |
| Wagle, 2017 | Wagle, 2017 | Wagle, 2017 |
| Wernicki et al. 2017 |  |  |
| Willison, 2019 | Willison, 2019 | Willison, 2019 |
| Wilson, 2018 |  |  |
| Zampara et al. 2017 | Zampara et al. 2017 |  |
| Zeng et al. 2010 | Zeng et al. 2010 | Zeng et al. 2010 |
| Ziegler, 2017 | Ziegler, 2017 | Ziegler, 2017 |

| **Step 4 (n=71)** | **Step 5 (n=46)** | **Step 6 (n=45)** | **Step 7 (n=27)** | **Step 8 (n=5)** |
| --- | --- | --- | --- | --- |
|  |  |  |  |  |
| Annamalai et al. 2013 | Annamalai et al. 2013 | Annamalai et al. 2013 |  |  |
|  |  |  |  |  |
|  |  |  |  |  |
|  |  |  |  |  |
| Baffoni et al. 2017 | Baffoni et al. 2017 | Baffoni et al. 2017 | Baffoni et al. 2017 |  |
| Bahrndorff et al. 2013 | Bahrndorff et al. 2013 |  |  |  |
| Balykina, 2019 |  |  |  |  |
|  |  |  |  |  |
|  |  |  |  |  |
|  |  |  |  |  |
| Branciari, 2016 | Branciari, 2016 | Branciari, 2016 |  |  |
| Buckley et al. 2010 | Buckley et al. 2010 | Buckley et al. 2010 |  |  |
| Bügener 2014 | Bügener 2014 | Bügener 2014 | Bügener et al. 2014 |  |
|  |  |  |  |  |
|  |  |  |  |  |
| Cean et al. 2015 | Cean et al. 2015 | Cean et al. 2015 |  |  |
| Chinivasagam et al, 2020 | Chinivasagam et al, 2020 | Chinivasagam et al, 2020 |  |  |
| Chintoan-Uta et al. 2016 | Chintoan-Uta et al. 2016 | Chintoan-Uta et al. 2016 |  |  |
|  |  |  |  |  |
|  |  |  |  |  |
| Clark et al. 2012 | Clark et al. 2012 | Clark et al. 2012 |  |  |
| Corrigan 2017 | Corrigan 2017 | Corrigan 2017 | Corrigan et al. 2017 |  |
| Currie et al. 2018 | Currie et al. 2018 | Currie et al. 2018 | Currie et al. 2018 | Currie et al. 2018 |
|  |  |  |  |  |
|  |  |  |  |  |
| Ebrahimi et al. 2015 | Ebrahimi et al. 2015 | Ebrahimi et al. 2015 | Ebrahimi et al. 2015 |  |
|  |  |  |  |  |
|  |  |  |  |  |
| El-Ghany et al. 2015 | El-Ghany et al. 2015 | El-Ghany et al. 2015 | El-Ghany et al. 2015 |  |
|  |  |  |  |  |
| Epps et al. 2015 | Epps et al. 2015 | Epps et al. 2015 | Epps et al. 2015 |  |
|  |  |  |  |  |
| Froebel et al. 2019 | Froebel et al. 2019 | Froebel et al. 2019 | Froebel et al. 2019 |  |
|  |  |  |  |  |
|  |  |  |  |  |
| Gaucher et al. 2015 |  |  |  |  |
|  |  |  |  |  |
| Gracia et al. 2016 A | Gracia et al. 2016 A | Gracia et al. 2016 A | Gracia et al. 2016 A |  |
| Gracia et al. 2016 B | Gracia et al. 2016 B | Gracia et al. 2016 B | Gracia et al. 2016 B |  |
|  |  |  |  |  |
| Guyard-Nicodème et al. 2017 | Guyard-Nicodème et al. 2017 | Guyard-Nicodème et al. 2017 | Guyard-Nicodème et al. 2017 | |
| Guyard-Nicodème et al. 2016 | Guyard-Nicodème et al. 2016 | Guyard-Nicodème et al. 2016 | Guyard-Nicodème et al. 2016 | Guyard-Nicodème et al. 2016 |
| Hammerl et al. 2014 | Hammerl et al. 2014 | Hammerl et al. 2014 |  |  |
|  |  |  |  |  |
| Haughton et al. 2013 |  |  |  |  |
|  |  |  |  |  |
|  |  |  |  |  |
|  |  |  |  |  |
|  |  |  |  |  |
| Hodgins et al. 2015 | Hodgins et al. 2015 | Hodgins et al. 2015 |  |  |
| Hovorková & Skřivanová, 2015 | |  |  |  |
| Huneay-Salaün et al. 2018 |  |  |  |  |
|  |  |  |  |  |
| Jansen et al. 2014 | Jansen et al. 2014 | Jansen et al. 2014 | Jansen et al. 2014 | Jansen et al. 2014 |
|  |  |  |  |  |
|  |  |  |  |  |
|  |  |  |  |  |
|  |  |  |  |  |
|  |  |  |  |  |
| Kelly et al. 2017 | Kelly et al. 2017 | Kelly et al. 2017 | Kelly et al. 2017 |  |
| Khan, 2019 |  |  |  |  |
| Khattak et al. 2018 | Khattak et al. 2018 | Khattak et al. 2018 | Khattak et al. 2018 | Khattak et al. 2018 |
| Kittler et al.. 2013 | Kittler et al.. 2013 | Kittler et al.. 2013 |  |  |
|  |  |  |  |  |
| Kobierecka et al. 2016 |  |  |  |  |
|  |  |  |  |  |
| Kurekci et al. 2014 | Kurekci et al. 2014 | Kurekci et al. 2014 | Kurekci et al. 2014 |  |
| Łaniewski et al. 2014 |  |  |  |  |
|  |  |  |  |  |
| Layton et al. 2011 | Layton et al. 2011 | Layton et al. 2011 |  |  |
| Liu, 2018 |  |  |  |  |
| Lourenco, 2019 |  |  |  |  |
|  |  |  |  |  |
|  |  |  |  |  |
|  |  |  |  |  |
| Massacci et al. 2019 | Massacci et al. 2019 | Massacci et al. 2019 | Massacci et al. 2019 |  |
|  |  |  |  |  |
|  |  | Meunier et al. 2017 |  |  |
| Meunier et al. 2017 | Meunier et al. 2017 | Moen et al. 2012 |  |  |
| Moen et al. 2012 | Moen et al. 2012 | Molatová et al. 2011 | Moen et al. 2012 |  |
| Molatová et al. 2011 | Molatová et al. 2011 |  | Molatová et al. 2011 |  |
|  |  |  |  |  |
| Mortada et al. 2020 |  |  |  |  |
|  |  |  |  |  |
|  |  |  |  |  |
|  |  | Nishii et al. 2015 |  |  |
| Nishii et al. 2015 | Nishii et al. 2015 |  | Nishii et al. 2015 |  |
|  |  |  |  |  |
|  |  | Nothaft et al. 2017 |  |  |
| Nothaft et al. 2017 | Nothaft et al. 2017 |  |  |  |
|  |  |  |  |  |
| Nuengjamnong & Luangtongkum 2014 | |  |  |  |
| Ocejo et al. 2017 |  |  |  |  |
|  |  |  |  |  |
| Park et al. 2017 |  |  |  |  |
|  |  |  |  |  |
|  |  |  |  |  |
| Prasai et al. 2016 |  |  |  |  |
| Ranjitkar, 2016 |  | Rashid et al. 2020 |  |  |
| Rashid et al. 2020 | Rashid et al. 2020 |  |  |  |
|  |  |  |  |  |
|  |  |  |  |  |
| Richards, 2019 |  |  |  |  |
|  |  | Saint-Cyr et al. 2017 |  |  |
| Saint-Cyr et al. 2017 | Saint-Cyr et al. 2017 |  |  |  |
|  |  |  |  |  |
|  |  |  |  |  |
|  |  |  |  |  |
|  |  |  |  |  |
|  |  |  |  |  |
|  |  |  |  |  |
|  |  | Schneitz and Hakkinen 2016 | |  |
| Schneitz and Hakkinen 2016 | Schneitz and Hakkinen 2016 | Skoufos et al. 2019 | Schneitz and Hakkinen 2016 | |
| Skoufos et al. 2019 | Skoufos et al. 2019 |  | Skoufos et al. 2019 | Skoufos et al. 2019 |
| Skånseng et al. 2013 | Skånseng et al. 2013 | Skånseng et al. 2013 | Skånseng et al. 2013 |  |
| Smialek et al. 2018 | Smialek et al. 2018 | Smialek et al. 2018 | Smialek et al. 2018 |  |
|  |  |  |  |  |
|  |  |  |  |  |
|  |  |  |  |  |
|  |  |  |  |  |
|  |  |  |  |  |
| Svetoch et al. 2011 | Svetoch et al. 2011 | Svetoch et al. 2011 |  |  |
|  |  |  |  |  |
| Szott et al. 2020 | Szott et al. 2020 | Szott et al. 2020 | Szott et al. 2020 |  |
|  |  |  |  |  |
| Theoret et al. 2012 | Theoret et al. 2012 | Theoret et al. 2012 |  |  |
| Thépault et al. 2020 |  |  |  |  |
| Thibodeau et al. 2014 | Thibodeau et al. 2015 | Thibodeau et al. 2015 | Thibodeau et al. 2015 |  |
| Thibodeau et al. 2015 |  |  |  |  |
| Thomrongsuwannakij et al. 2016 | |  |  |  |
|  |  |  |  |  |
|  |  |  |  |  |
| Tsiouris, 2018 |  |  |  |  |
|  |  |  |  |  |
| Valečková et al. 2020 |  |  |  |  |
|  |  |  |  |  |
| Vandeputte, 2019 |  |  |  |  |
| Visscher, 2019 |  |  |  |  |
|  |  |  |  |  |
| Wagle, 2017 | Wagle, 2017 | Wagle, 2017 |  |  |
|  |  |  |  |  |
|  |  |  |  |  |
|  |  |  |  |  |
|  |  |  |  |  |
| Zeng et al. 2010 |  |  |  |  |
| Ziegler, 2017 | Ziegler, 2017 | Ziegler, 2017 | Ziegleret al. 2017 |  |
|  |  |  |  |  |
|  |  |  |  |  |
|  |  |  |  |  |
