# Supplementary material for: Systematic review of products with potential application for use in the control of Campylobacter spp. in organic and free-range broilers
Source: Acta Vet Scand. 2022 Sep 8;64:24. doi: 10.1186/s13028-022-00644-z (PMC9461118; doi:10.1186/s13028-022-00644-z)
Supplement: Supplementary file 5 — Additional file 5. Reduction of Campylobacter. List of articles before the final step in the review process (Fig. 1), the tested interventions and the effect of the interventions. [file 13028_2022_644_MOESM5_ESM.docx]

|  |  |  |  |  |
| --- | --- | --- | --- | --- |
| **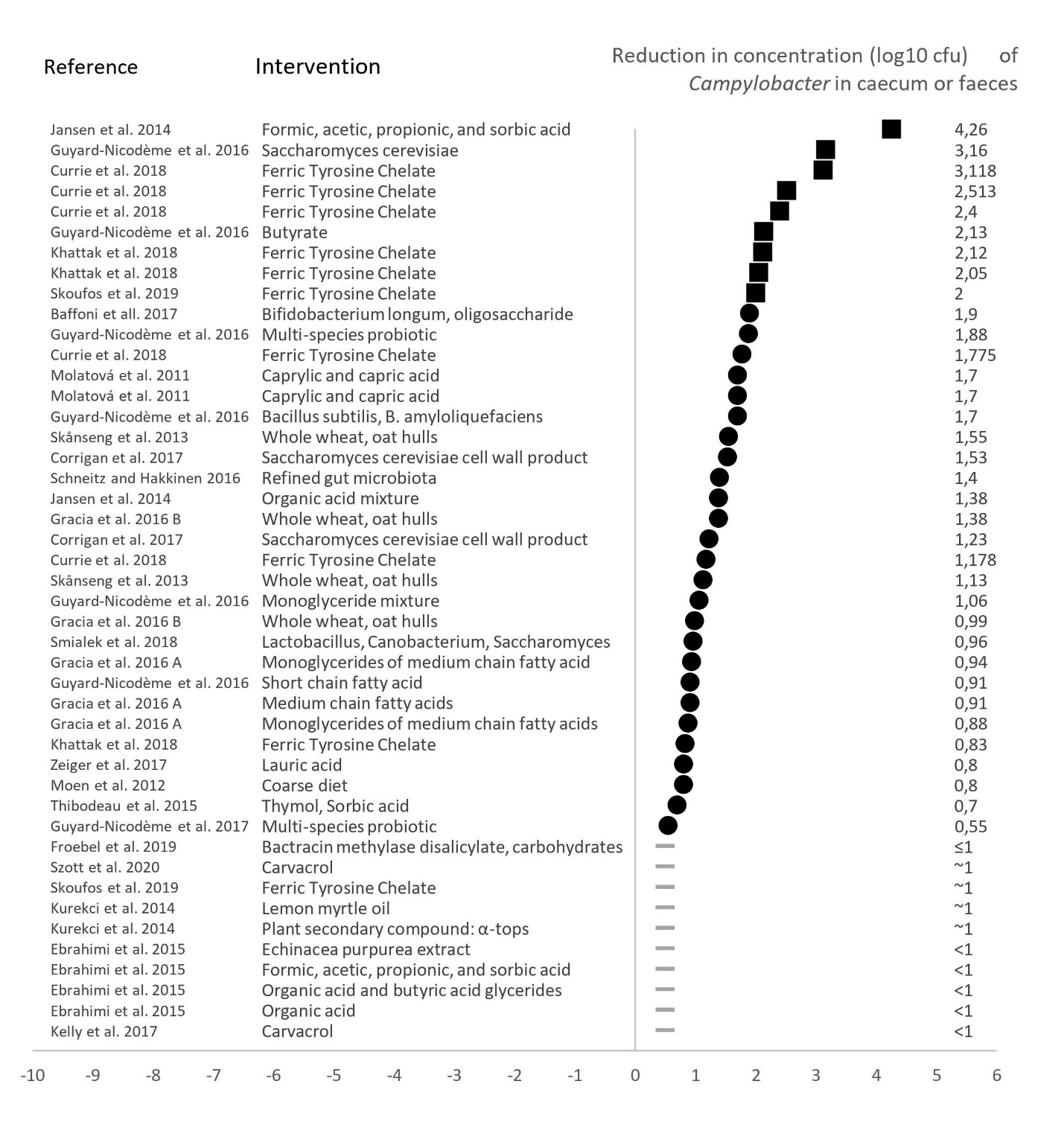** |  |  |  |  |

**Additional file 5. Reduction of Campylobacter in the found studies before the final step in the review process**

Overview of studies included (black squares) and excluded (black dot and grey line) in the final step of the screening of the literature review. Grey lines indicate studies that did not provide exact values of the reduction. Some studies are listed more than once if the study tested more than one product or tested different interventions with the same product. Four excluded studies (Bügener et al. 2014, El-Ghany et al. 2015, Massacci et al. 2019, Nishii et al. 2015) are not listed as it was not possible to estimate a log10 reduction from the results.
